# Biomechanical considerations in the design of patient-specific fixation plates for the distal radius

**DOI:** 10.1007/s11517-018-1945-6

**Published:** 2018-12-26

**Authors:** G. Caiti, J. G. G. Dobbe, E. Bervoets, M. Beerens, S. D. Strackee, G. J. Strijkers, G. J. Streekstra

**Affiliations:** 1 Amsterdam UMC, University of Amsterdam, Biomedical Engineering and Physics, Amsterdam Movement Sciences, Meibergdreef 9, Amsterdam, Netherlands; 2 Metrotech Engineering & Physics, Begtrupvej 75, Sporup, Denmark; 3 Xilloc Medical B. V., Urmonderbaan 22, Geleen, Netherlands; 4 Amsterdam UMC, University of Amsterdam, Plastic Reconstructive and Hand Surgery, Amsterdam Movement Sciences, Meibergdreef 9, Amsterdam, Netherlands

**Keywords:** Corrective osteotomy, Volar distal radius plate, Patient-specific implant, Implant design, Finite element analysis

## Abstract

Use of patient-specific fixation plates is promising in corrective osteotomy of the distal radius. So far, custom plates were mostly shaped to closely fit onto the bone surface and ensure accurate positioning of bone segments, however, without considering the biomechanical needs for bone healing. In this study, we investigated how custom plates can be optimized to stimulate callus formation under daily loading conditions. We calculated implant stress distributions, axial screw forces, and interfragmentary strains via finite element analysis (FEA) and compared these parameters for a corrective distal radius osteotomy model fixated by standard and custom plates. We then evaluated these parameters in a modified custom plate design with alternative screw configuration, plate size, and thickness on 5 radii models. Compared to initial design, in the modified custom plate, the maximum stress was reduced, especially under torsional load (− 31%). Under bending load, implants with 1.9-mm thickness induced an average strain (median = 2.14%, IQR = 0.2) in the recommended range (2–10%) to promote callus formation. Optimizing the plate shape, width, and thickness in order to keep the fixation stable while guaranteeing sufficient strain to enhance callus formation can be considered as a design criteria for future, less invasive, custom distal radius plates.

**Graphical abstract:**
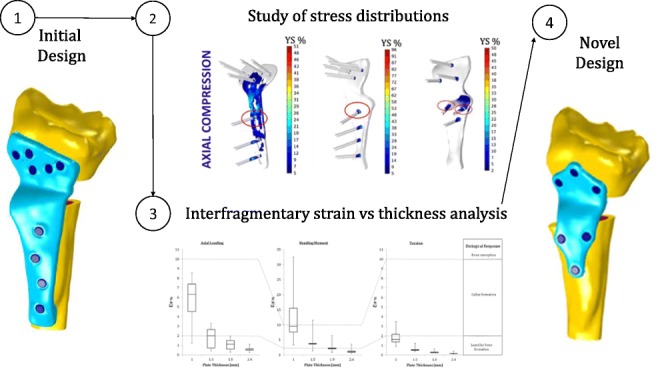
ᅟ

ᅟ

## Introduction

Malunion of the distal radius is the most common complication arising after the suboptimal healing of a distal radius fracture [1]. Depending on the amount of deformity, the malunion can seriously compromise the natural biomechanics of the wrist joint, inducing early osteoarthritis and pain. Symptomatic cases, occurring in approximately 5% of all the distal radius fractures [2], are surgically treated by corrective osteotomy. The aim of corrective osteotomy is to restore the anatomical alignment of the bone segments with subsequent plate fixation. In order to achieve good functional results, an accurate positioning of the radius segments is fundamental [3].

Volar anatomical plates, shaped to follow the average patient anatomy, should facilitate fracture reduction. Nonetheless, when the bone anatomy is deformed due to a malunion, standard anatomical plates do not fit properly and fracture reduction highly depends on the experience and skills of the surgeon to estimate how to manually bend the implant to achieve adequate fixation [4].

A promising treatment option to ensure accurate bone repositioning is the use of patient-specific plates, designed to fit the individual patient’s bone anatomy and to fixate the bone segments according to a preoperative plan [5, 6]. However, in these personalized distal radius plates, parameters such as implant screw configuration, size, and thickness which affect the mechanical characteristics of the implant have remained essentially similar to standard anatomical plates.

Fixation by highly rigid plates can be disadvantageous. A consequence of too rigid fixation is that the plate carries the vast majority of the load, leaving the bone segments deprived of the mechanical stimuli needed to maintain bone mass and to promote posttraumatic osteogenesis [7]. Frequently reported consequences of this stress shielding include bone resorption, deficient callus formation, delayed union, late implant failure, and non-union [8]. It is known that bone healing by callus formation, referred to as secondary bone healing, is stimulated by moderate interfragmentary movements [9]. Based on Perren’s theory [10], strain has an effect on the tissue differentiation during the fracture healing process. Without tissue strain in the fracture gap, there is no mechanical induction of callus formation. With strain values up to 2%, direct bone healing with lamellar bone formation occurs. Strain values between 2 and 10% induce callus formation and are tolerated by the three-dimensional configuration of newly forming bone tissue (woven bone). However, when the strain reaches higher values than 10%, bone resorption prevails and bone bridging does not occur [10, 11].

In order to enhance secondary bone healing and prevent bone resorption, a type of fixation is required that generates the required amount of strain in the newly formed bone tissue by allowing controlled interfragmentary movements along the bone’s axial direction [7].

An efficient way to analytically simulate fracture-healing with different fixation strategies is computational modeling. Finite element analysis (FEA) studies have already provided insight in the mechanical characteristics of custom implants for a number of orthopedic and orthodontic applications [12, 13].

In this paper, we use FEA in order to determine to which extent a patient-specific plate for the radius can be optimized for stress within the implant and strain within the gap, by varying screw configuration, plate size, and plate thickness.

## Methods

### Custom plate design

**Fig. 1:**
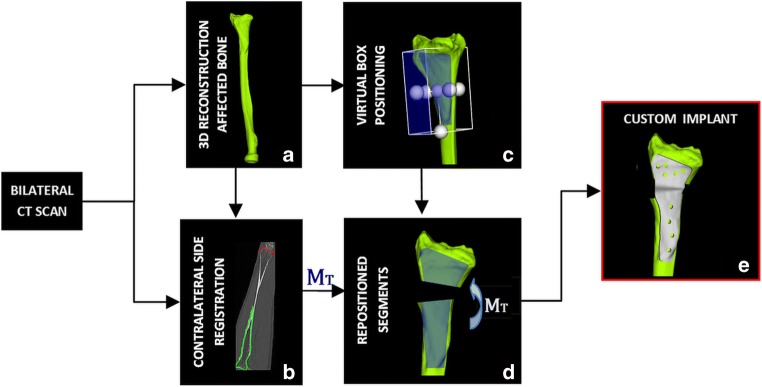
Steps for preoperative planning and design of a custom implant. **a** 3D reconstruction of the affected bone. **b** Distal and proximal parts of the affected bone model registered onto the mirrored image of the healthy contralateral radius. **c** Virtual box used to select the target bone surface for the implant design. **d** Selected bone surface extruded into a 3D plate, cut and then repositioned via the transformation matrix (M_T_). **e** Repositioned parts of extruded plate footprint with interpolated connection

Preoperative 3D surgical planning and patient-specific implant design were performed with custom-made software, previously described by Dobbe et al. [5]. Briefly, planning and design of the implants was as follows: as a starting point, we obtained bilateral high-resolution CT scans of both radii of five patients using a clinical protocol (Philips Brilliance 64 CT scanner, Cleveland, OH; voxel size 0.45×0.45×0.45 mm, 120 kV, 150 mAs, pitch 0.6). The 3D model of the malunited radius was obtained by segmentation using a level set-based algorithm initialized by region growing (Fig. 1a).

Distal and proximal bone segments of the affected radius model, excluding the deformity, were registered to the mirrored image of the contralateral bone by point-to-image registration to find the optimal required anatomical alignment. The result of the registration was a correction matrix M_T_ (Fig. 1b) which describes the movement of the distal bone segment to the planned position. In order to design the plate, a virtual box was interactively positioned to enclose the target surface on the affected bone polygon (Fig. 1c). Subsequently, one selected face of the virtual box was projected onto the bone polygon, resulting in the implant footprint. The projected surface was then extruded to create a temporary plate, fitting the affected bone surface. Next, the affected bone polygon and the temporary plate polygon were cut by a user-defined virtual cutting plane and repositioned with the correction matrix (M_T_) in 3D space (Fig. 1d). The missing piece between the two plate segments was created by Bezier interpolation between corresponding points (Fig. 1e).

For modeling screws, threads were neglected since global stress values on plate and screws are not affected by local mechanical responses at the interface between thread and bone, as also reported by other previously published FEA studies on orthopedic implants [13, 14].

### Finite element modeling

Three-dimensional models of bones and osteosynthesis material (implants and screws) were meshed with tetrahedral mesh elements using COMSOL Multiphysics (Version 5.3, COMSOL Inc., Burlington, MA).

A convergence test was run to assess the mesh refinement level. The aim of the test was to investigate for which tetrahedron element sizes the von Mises stress is independent on the mesh size. This is evaluated in an implant region of high curvature, i.e., at screw locations.

Mechanical material properties for plates and screws were chosen in agreement with commercially available volar distal radius plates (2.4-mm LCP Volar Column Distal Radius Plate, Synthes, West Chester, PA) and accompanying locking screws (2.4-mm locking screws, Synthes, West Chester, PA). The custom distal radius plates were assumed to consist of titanium alloy Ti-6Al-4V (Young’s modulus *E* = 110 GPa, and Poisson’s ratio *ν* = 0.35). Implant screws were assumed to consist of unalloyed titanium Ti-Grade 4 (*E* =109 GPa, *ν* = 0.33). Model parameters for bone layers were *E* = 17 GPa, *ν* = 0.33, for the external cortical layer and *E* = 13 GPa, *ν* = 0.3 for the internal cancellous layer [14]. The thickness of the bone layers was extracted from the CT scan with the previously described software, by separately segmenting the cortical and cancellous bone. All the materials were considered isotropic and linearly elastic. The connection between the plate and the screws was modeled as a union of the objects. Since locking screws were modeled, the contact between the screws and the plate was considered adhesive.

### Loading conditions

**Fig. 2:**
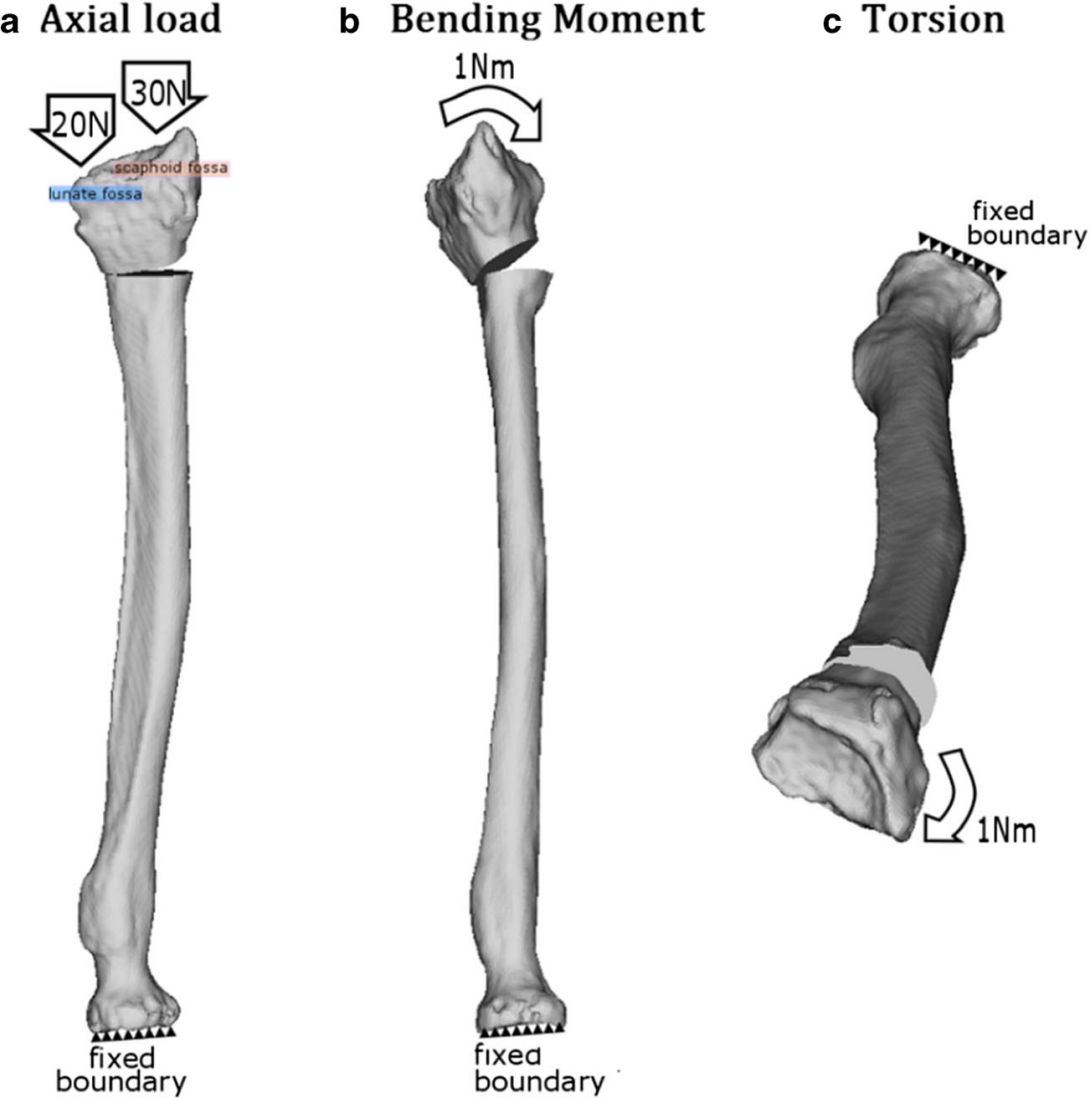
Definition of daily loading conditions. **a** Forty percent of the axial load was exerted onto the lunate and 60% onto the scaphoid fossa. **b** Bending moment about the axis defining wrist flexion-extension motion. **c** Torsion

To simulate forces acting on the radius and the osteosynthesis material during daily activity in subjects who underwent reconstructive surgery, we separately applied three loading conditions to each radius model geometry, viz. axial compression (Fig. 2a), bending (Fig. 2b), and torsion moments (Fig. 2c) [14]. In vivo loading conditions in the human distal radius are not completely known. Bernal et al. measured the grip force in different subjects through wearable capacitive pressure sensors in the fingers while performing a daily-life activity such as lifting a half-full 1-l bottle. The mean grip strength was 18.6 N [15]. Since every 10 N of grasp force transmits 26.4 N through the radius in the axial direction [16], the axial compression is approximately 49 N. Based on these estimates, we used 50 N for axial compression. Axial forces were distributed through the radius over the scaphoid fossa (60%) and over the lunate fossa (40%). Magnitude of bending and torsion moments applied was 1 Nm [14].

### Definition of plate design criteria

A main factor in plate fixation is the maximum axial pulling force exerted on the screws. In this study, the screw pull-out force was considered 148 N per millimeter of cortical layer, as experimentally obtained by Lyon et al. [17]. Therefore, the threshold for axial force per screw was set to 444 N (148 N holding power ×1.5 average millimeter thickness of cortical layer of the bone model ×2 cortex layers engaged by the screw). The allowable maximum stress in the implant was set to the plastic deformation threshold (yield stress YS) of the osteosynthesis materials (YS = 1060 MPa for the Ti-6Al-4V plate and YS = 400 MPa for the Ti-Grade 4 screws).

**Fig. 3:**
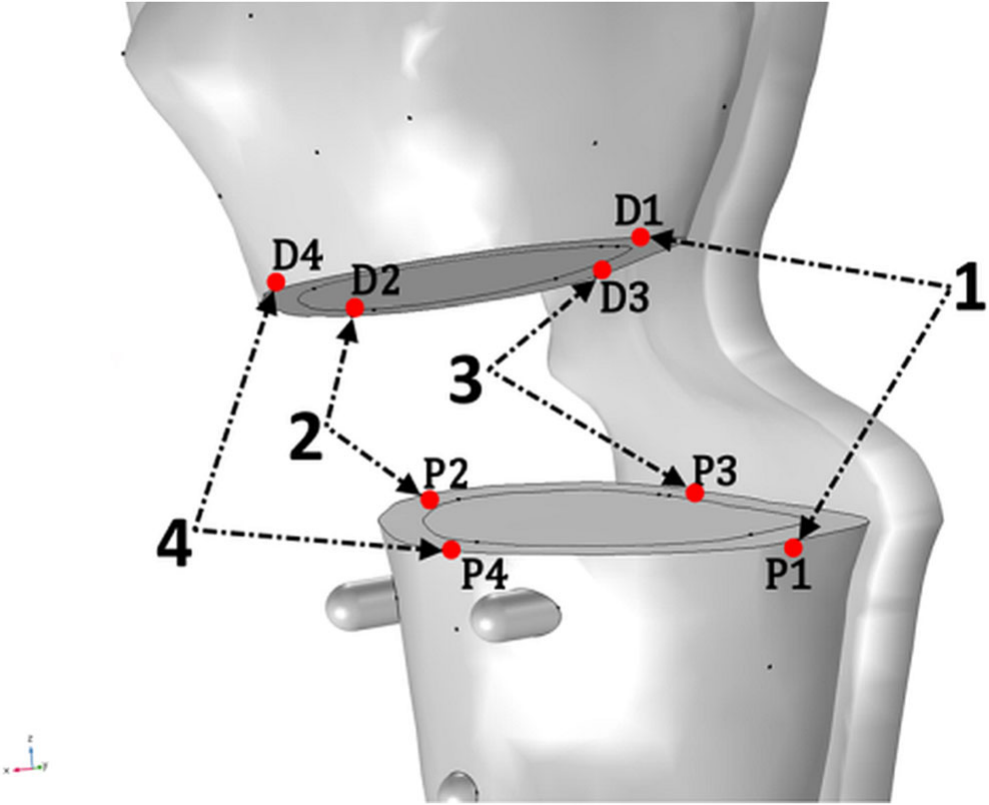
Definition of corresponding distal and proximal point pairs (*D*_*i*_, *P*_*i*_) at the osteotomy border on the distal and the proximal bone segments used to calculate the average strain in the gap

The interfragmentary strain (*ε*_*IF*_) depends on the relation between the dimension of fracture gap (*L*) and the change in *L* due to loading (*δL*): ${\varepsilon}_{IF}=\frac{\delta L}{L}$ [10]. Strain ranging from 2 to 10% is considered optimal for callus formation [10]. We estimated the strain (*ε*_*IF*_) as the mean relative change in distance between four different corresponding node pairs (*D*_*i*_, *P*_*i*_) when load was applied to the bone model. Node pairs were selected uniformly across the perimeter of the bone cross-section in the osteotomy cutting plane (Fig. 3):

### Stress distribution in standard and custom plates

**Fig. 4:**
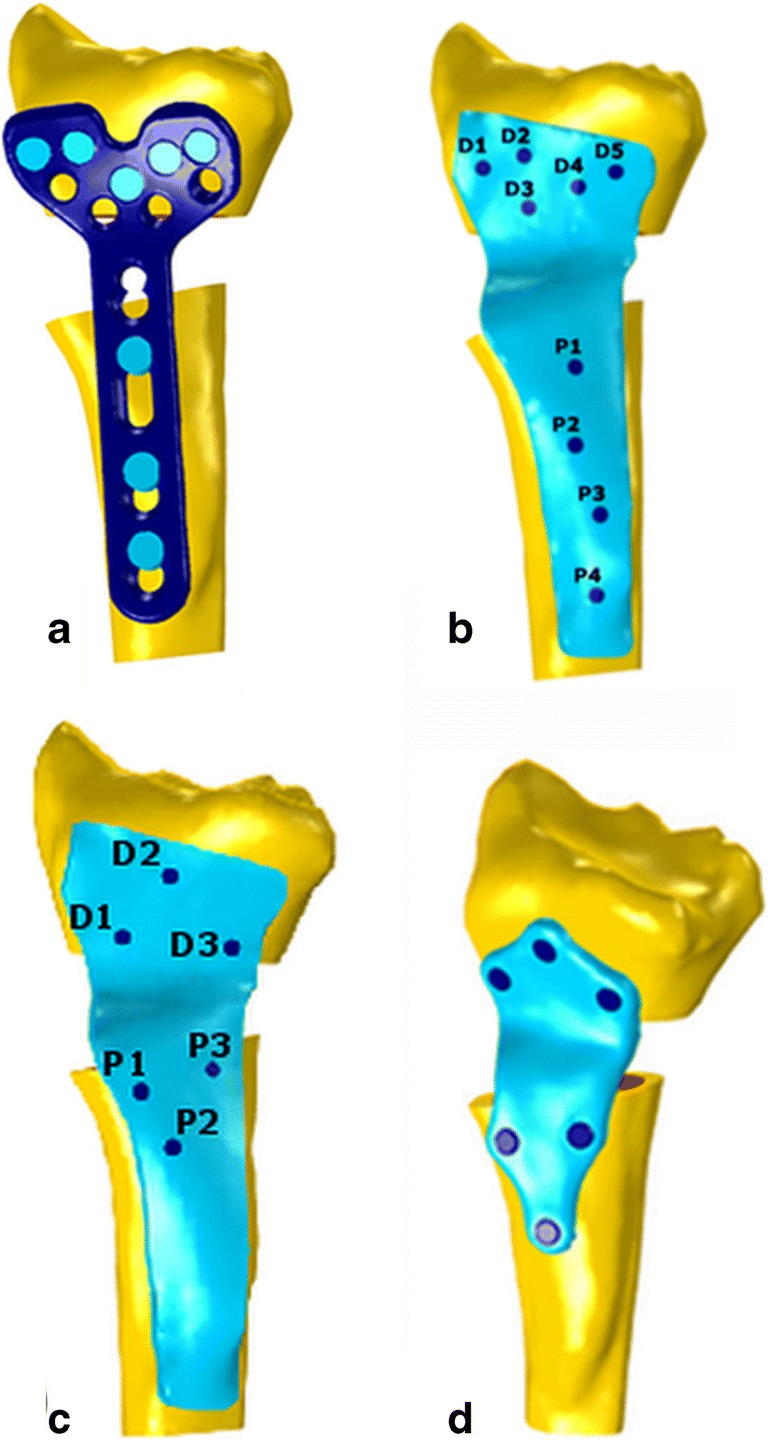
Implant types: **a** standard anatomical plate (2.4-mm LCP Volar Column Distal Radius Plate, Synthes, West Chester, Pennsylvania, USA). **b** Initial custom implant configuration. Five screw holes were positioned distally (D1, D2, D3, D4, D5) and four proximally (P1, P2, P3, P4). **c** Custom implant with the new screw configuration. **d** Custom implant with the new screw configuration and a reduced plate size

In order to compare mechanical performance of the custom plate with its standard counterpart, the stress distribution in the plate and strain in the gap were estimated for a standard volar locking compression plate (LCP) currently used at our institution (2.4-mm LCP Volar Column Distal Radius Plate, Synthes, West Chester, Pennsylvania, USA) (Fig. 4a). Since the standard implant shape generally does not fit a malunited bone, in the simulation, we positioned the implant as close as possible to the bone, as would be the case in actual surgery. One of the four proximal screws was not used in the standard plate because it protruded into the gap. Stress distributions in the plate, gap strains, and axial screw forces were calculated for custom plates under the same loading conditions. As a starting point for the analysis, we considered a custom plate of size and screw configuration similar to the standard anatomical distal radius plate. We will refer to it as the “initial custom plate” (Fig. 4b). Nine screw holes (orientation perpendicular to the bone surface, 1.8 mm core diameters) were added to the plate. Five screws were positioned on the distal and four on the proximal part of the plate. The initial plate thickness was set to 2.4 mm.

### Definition of an improved screw pattern

Results of this first analysis were used in order to decide if there were redundant screws and/or if the screws could have been placed in a more efficient position in the initial custom plate. When an improved screw configuration was found, the shape of the plate was changed accordingly.

### Reducing the custom implant thickness

In the second set of simulations, the improved screw pattern and plate shape were applied to four additional patient cases. In each case, a virtual corrective osteotomy was performed with the procedure previously described.

Four different plate thicknesses (2.4, 1.9, 1.5, and 1.0 mm) were applied to the considered plate models. Gap strain and stress distribution in the plates were estimated in each case.

## Results

### Convergence test

Three meshing schemas were evaluated on the same bone-implant model, with different tetrahedron sizes (Table 1). Relative errors in Von Mises stress, as measured in the area of interest, were lower than 0.1% for mesh sizes Normal and Fine. In order to maintain a good balance between computational time and mesh refinement, the scheme “Fine” was applied in all subsequent evaluations.

**Table 1:** Element size in the mesh models used for FEA, average element volume, and relative error in the measured Von Mises stress

	Coarse	Normal	Fine
Number of elements	418,732	462,446	531,178
Average element volume [mm^3^]	50 × 10^−3^ ×10^−3^	45 × 10^−3^ × 10^−3^	39 × 10^−3^ × 10^−3^
Relative error %	0.35	0.02	0.02

### Stress distribution in standard and custom plates

**Fig. 5:**
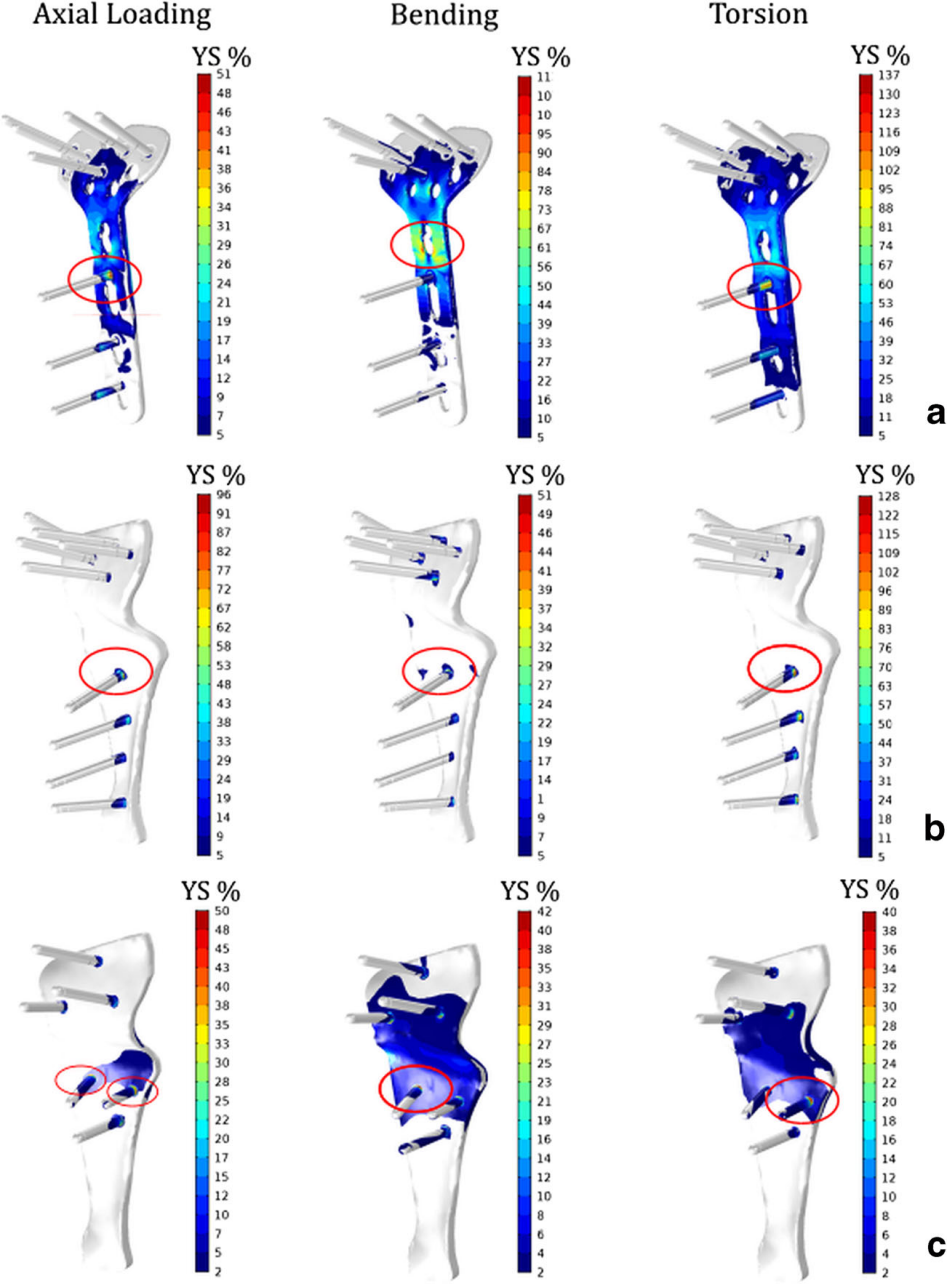
**a** Color maps showing Von Mises stress distribution under axial compression (50 N), bending moment (1 Nm), and torsion (1 Nm). Stresses are expressed as a percentage of the yield stress of the compounding materials. Location of the highest stress, as indicated by each applicable color scale, is also red circled. Regions with stress below the minimum value along the scale are colored white. **a** Standard plate. **b** Initial custom plate. High stress concentrations were observed at the level of the proximal screw closer to the gap. **c** Custom plate with modified screw configuration

The average Von Mises stress distributions in the standard plate are presented in Fig. 5a. Stresses are expressed as a percentage of the yield stress (YS) of the materials present in the osteosynthesis materials. In the standard plate, the maximum stress exceeded the YS under bending at the osteotomy site and under torsion at the level of the proximal screw closer to the gap*.* In the initial custom plate, the highest stress concentration occurred at the level of the proximal screw close to the osteotomy gap (P1), in all three load cases, Fig. 5b. The stress exceeded the YS at the plate-screw boundary at P1 during torsional loading.

### Definition of an improved screw pattern

The measured axial screw forces in the initial custom plate (Table 2) were far below the threshold of 444 N.

**Table 2:** Axial screw forces in the initial and modified custom plate configurations under the three daily load conditions assumed. D1–D5 represent distal screws; P1–P4 represent proximal screws. All reported values are far below the pull-out threshold of 444 N

Custom Plate Type	Load	Axial Screw Force [N]								
		D1	D2	D3	D4	D5	P1	P2	P3	P4
Initial	Axial	− 28.6	− 11.9	− 9.7	21.8	31.5	− 33.3	50.1	− 74.0	27.4
	Bending	27.6	95.4	− 130.8	− 34.5	42.7	− 85.4	64.6	− 1.4	3.0
	Torsion	− 50.9	− 8.8	− 23.6	36.8	41.1	− 27.9	92.5	− 113.1	31.4
Modified	Axial	− 46.0	− 2.5	42.2	–	–	31.8	16.1	− 67.9	–
	Bending	− 67.8	115.0	− 44.6	–	–	− 52.6	120.6	− 65.6	–
	Torsion	− 85.7	12.0	79.7	–	–	135.1	− 38.3	− 102.3	–

These results suggested that the screw configuration can be optimized by removing redundant screws and placing more screws in the area with higher stress in the plate. In the initial custom plate geometry, the four proximal screws were directed toward the bone axis with insufficient lever to counteract the exerted torsional moment. We improved this initial configuration by placing three distal and proximal screws in a triangular pattern (Fig. 4c).

**Fig. 6:**
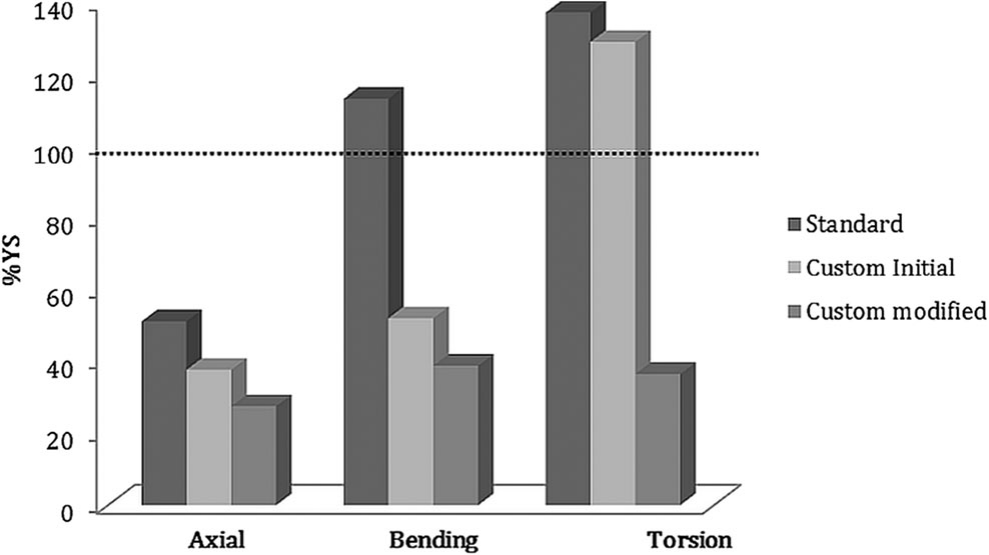
Maximum Von Mises stress value, in percent of yield stress (YS) for implant-screw assembly under axial compression (50 N), bending moment (1 Nm), and torsion (1 Nm) in the standard, initial, and modified custom plates

Figure 5c represents the Von Mises stress distribution in the custom plate with the modified screw configuration. The highest stress region shifted from the single proximal screw closer to the gap in the standard and initial custom plate to the two parallel screws in the newly configured plate. Comparison between the maximum value of Von Mises stresses measured in the standard, initial, and modified custom plates is displayed in Fig. 6. The plastic deformation limit was exceeded under bending moments and under torsion for the standard plate and under torsion by the initial custom plate. For the modified custom plate, on the other hand, the peak stresses were in the acceptable range in all the load cases.

Screw pull-out forces (Table 2) remained below the threshold.

Since the tail of the custom plate was bearing almost no stress, the implant was resized while maintaining a space of at least 1.5 × the screw diameter around each screw (Fig. 4d).

Table 3 shows the percentage of interfragmentary strain *ε*_*IF*_ experienced with the standard, initial and modified geometry of the custom plate. Both custom implant configurations did not facilitate adequate interfragmentary strain to promote secondary healing at a plate thickness of 2.4 mm. Strain values induced by standard plate fixation were in the desired range under a bending moment.

**Table 3:** Average strain % in the initial custom plate configuration and in the modified custom plate under axial compression (50 N), bending moment (1 Nm), and torsion (1 Nm). For custom implants (thickness 2.4 mm), the gap strains were not in the desired range for callus formation (2–10%) in any of the load cases

Load	*ε*_*IF*_%		
	Standard	Initial custom	Modified custom
Axial compression	0.3	0.1	0.2
Bending	4.3	1.1	1.3
Torsion	0.5	0.1	0.1

### Reducing the custom implant thickness

**Fig. 7:**
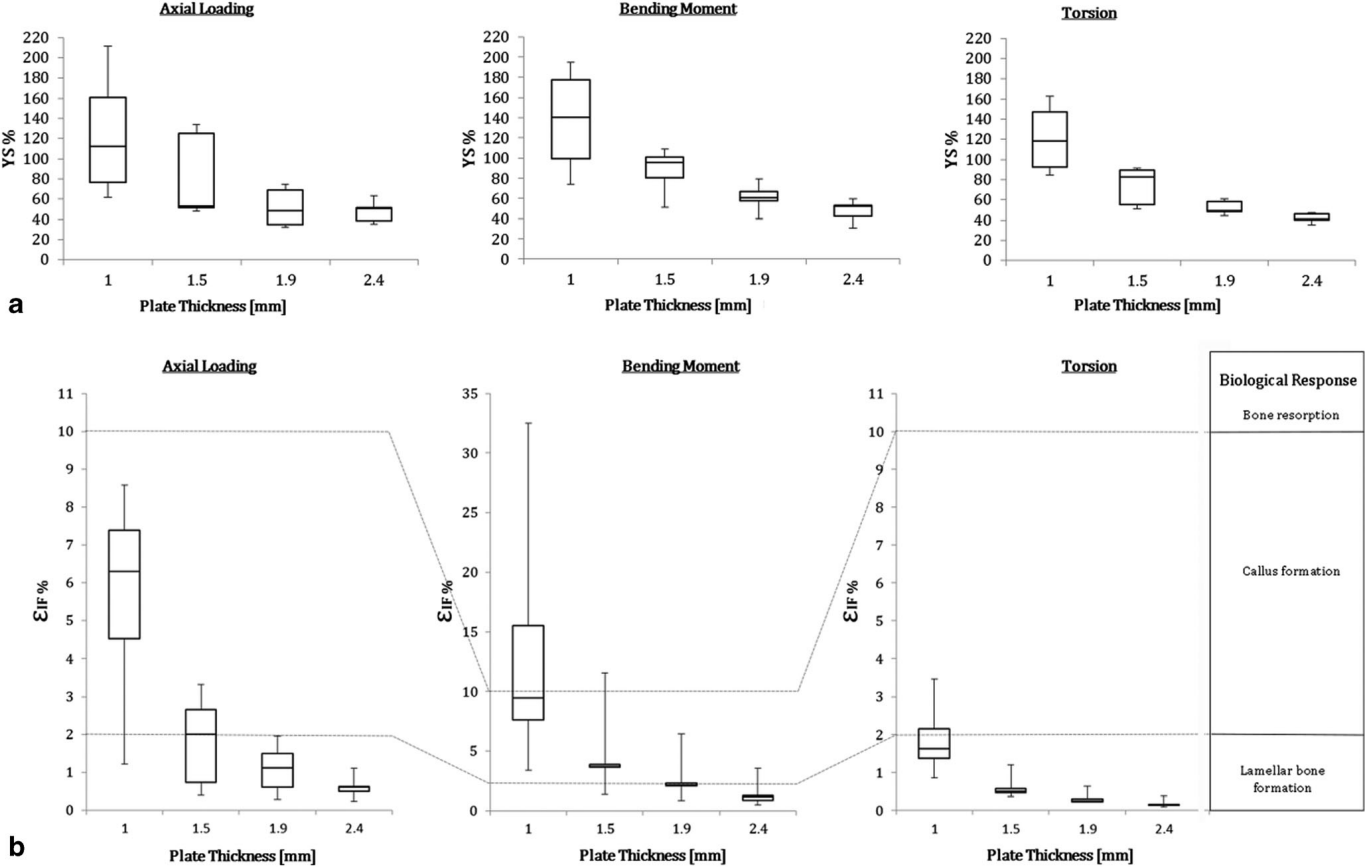
Boxplots representing **a** the distribution of maximum Von Mises stress and **b** distribution of the gap interfragmentary strain *ε*_*IF*_ in five patient cases as a function of the modified custom plate thickness (2.4, 1.9, 1.5, and 1.0 mm) under axial compression (50 N), bending moment (1 Nm), and torsion (1 Nm). Based on Perren’s theory, each strain range induces a different biological response

Figure 7a shows the distribution of the maximum Von Mises stresses in the modified custom plate at different thicknesses, for five patient cases. As expected, reduction of the plate thickness generated an increase in the maximum stress. Maximum stress remained below YS for an implant thickness of 1.9 mm for all the selected patient cases.

Reduction of the modified plate thickness resulted in an increase in the interfragmentary strains. Figure 7b shows the distribution of the strain values measured in the five patient cases with modified custom plates at different thicknesses. Adequate strain was achieved under bending (in the range 2–10%) with 1.5- and 1.9-mm-thick plates.

## Discussion

With the recent developments in 3D printing technology, the use of patient-specific instruments and implants in orthopedic surgery is becoming increasingly popular [18]. In the field of corrective osteotomy of the distal radius, it has been demonstrated quantitatively that the use of custom plates, matching the anatomy of the bone segments and bridging them to the planned position, allows accurate anatomical limb reconstruction in six degrees of freedom [5].

Conventionally, the defect in corrective osteotomy of the radius is filled using a graft before fixation. However, bone grafting can lead to donor-side morbidity, delayed healing, and inaccurate reconstruction due to a mismatch between the graft and the gap size. A current trend is therefore to fixate without bone grafting. Recent studies on large patient cohorts showed that bone grafting can often be omitted and it is not mandatory for bone healing and fracture displacement prevention [19]. In the simulated osteotomies reported in this study, we followed the current trend to perform corrective osteotomy without bone grafting and used gap heights and gap volumes (Table 1) in agreement with those reported in clinical studies on this particular topic [19].

Our study showed that under daily loads, the stress in the standard anatomical plate exceeds the yield stress, which may therefore lead to implant failure. In the initial patient-specific plate, peak stresses under daily-loading conditions were generally lower compared to the standard anatomical plate, although the peak stress under torsion also exceeded the yield stress for the proximal screw close to the gap.

By applying an alternative screw configuration in the modified custom plate, with six screws arranged in triangular patterns in the distal and proximal segments, the lever arm that counteracts the torsional moment was increased, reducing the peak stress in the plate by 31%. By minimizing the plate size and the number of screws, the risk of screw protrusion and related complications such as intra-articular damage, flexor tendon irritation, and/or neurovascular compromise can be reduced [20].

As was expected, the gap strain increased by reducing the plate thickness. Implants having a thickness of 1.9-mm thickness yielded an average level of strain in the optimal range for callus formation (median = 2.14% IQR = 0.2) under a bending load, while the maximum stress in the plate remained below 82% of the yield stress. The modified custom plate therefore acts as an “internal fixator,” splinting the bone segments and providing a flexible fixation strategy rather than compression [10].

Although we modeled the cortical and trabecular bone differently, it was still a simplification of the actual mechanical properties of healthy bone tissue, which may therefore be considered a limitation of our study. We further considered the bone uniform and linearly elastic, which is a limitation of the study since it is well recognized that bone material is orthotropic and inhomogeneous [21, 22]. By not including the orthotropic characteristic of the bone, we introduced an error of approximately 5% while not including inhomogeneity in our model may generate a larger error up to 20% [21, 22]. However, we expect that even an underestimation of the strain of the 20% would merely shift the median of our observations within the biological response ranges, without changing the conclusion of the study.

For future studies, we recommend including patient-specific bone inhomogeneity and anisotropy in the model. This may be especially of interest in cases of bone degradation (e.g., due to osteoporosis), which may affect screw pullout strength [23].

## Conclusion

In conclusion, placing the fixation screws in a triangular configuration in custom distal radius plates as proposed reduces the maximum stress compared to a standard screw configuration, which helps reducing the size of the implant. Choosing the optimal plate thickness further improves customizing osteosynthesis by allowing sufficient strain to enhance callus formation. Future mechanical evaluation is needed to experimentally confirm these findings.
